# Editorial: Executive Function(s): Conductor, Orchestra or Symphony? Towards a Trans-Disciplinary Unification of Theory and Practice Across Development, in Normal and Atypical Groups

**DOI:** 10.3389/fnbeh.2018.00085

**Published:** 2018-05-08

**Authors:** Lynne A. Barker, Nicholas Morton

**Affiliations:** ^1^Reader in Cognitive Neuroscience, Brain, Behaviour and Cognition Group, Department of Psychology, Sociology and Politics, Sheffield Hallam University, Sheffield, United Kingdom; ^2^Consultant Clinical Neuropsychologist, Neuro-Rehabilitation Services, Rotherham & South Humber NHS Trust, Tickhill Road Hospital, Doncaster, United Kingdom

**Keywords:** executive functions, development, psychometrics, imaging, three-dimensional, neuropathology

One problem with well-established executive function theories is that developmental disorders, brain injury, neuropathology, psychiatric conditions, and cognitive decline typically produce cross-cutting problems in social, cognitive, and emotional domains that seldom correspond to executive function models. Consequently, there is an argument that conceptual theories of executive function do not accord with clinical presentation (Manchester et al., 2004), and that executive function tests have limited predictive clinical utility (Barker et al., 2004; Burgess et al., 2006). Currently, there is need for unification of executive function approaches across disciplines, populations, and life span, further, it is also necessary for narrowing the conceptual gap between theoretical positions, clinical symptoms, and measurement.

This research topic includes findings on the development of executive functions in childhood, adolescence, and early adulthood. Taylor et al. found that the executive functions developed non-linearly in late adolescence and early adulthood, with peaks and troughs in executive ability corresponding to morphological brain change at these age ranges. These findings have ramifications for understanding the normal and abnormal development of executive functions. The reviewed evidence also indicates that working memory, attention, and inhibitory control develop alongside time keeping skills and may depend upon shared but distinct neural substrates (Vicario).

One possibility is that cognitively controlled timing skills make some unique but distinct contribution to the development of working memory and executive functions. Hsu et al. reviewed studies on the development and malleability of Executive Control (EC), which is defined as capacity to regulate cognitive processes for successful goal attainment. They concluded that targeted EC training interventions would likely benefit children from low socio-economic status backgrounds and those with attention-deficit disorders, although early findings are insufficient to warrant firm conclusions. The use of new neuroimaging techniques and better understanding of mechanisms underpinning EC training could further inform developmental interventions for targeted populations.

This special edition includes evidence that performance on supposed executive function and memory measures may depend upon some shared process defined as *fluid intelligence*. Royall and Palmer used a latent variable structural equation modeling approach to distinguish domain-specific variance in executive function and memory measures and shared cognitive variance defined by Spearman's *g* (where *g* represents general intelligence). When variance was accounted for across several memory and executive function measures, executive function ability overlapped with intelligence scores in a healthy elderly cohort. These findings have implications for classification and specificity of executive functions, measurement, and assessment and purported neural substrates. Similarly, impaired performance on a battery of executive function tests by schizophrenic patients was mostly explained by deficits in fluid intelligence Roca et al. Importantly, when fluid intelligence was partialled out multitasking and decision-making performance deficits remained indicating selective executive in addition to general cognitive deficits.

Executive attentional control and working memory functions have been investigated in a range of psychiatric and neuropathological conditions. Drabble et al. investigated the potential role of attentional control to self-harm in people with borderline personality disorder (BPD). However, the findings were surprising, high attentional focusing predicted self-harm history in those with high BPD features. In contrast, good attentional switching ability reduced the likelihood of self-harm. The notion of a potential moderating effect of attentional control on negative affect and self-harm in BPD individuals constitutes a new conceptualization of the condition. The review of extant evidence indicated that basal forebrain cholinergic system is a potential neural basis of executive attention (Villano et al.). Collectively, data supported the notion that neuropeptide regulatory orexin neurons stimulate cholinergic frontal pathways and may provide the mechanism of executive attention, but a further detailed work is needed.

Working memory capacity was also investigated in BPD individuals using an event related potentials (ERP) paradigm. Liu et al. found that BPD patients had lower P3 amplitudes and longer N2 latencies than controls that were independent of working memory load likely indicating working memory dysfunction. Working memory abnormalities were also found in mild cognitive impaired (MCI) patients based on distant synchronization of the background network at rest and during working memory task performance (Wang et al.). There was no significant difference between rest and working memory state in MCI patients when compared to controls indicating inefficient organization of the background network associated with cognitive impairment in patients. The neural locus of working memory networks was also explored in bilateral dorsolateral prefrontal cortices using a resting state functional connectivity approach mapped to working memory task accuracy in healthy controls (Fang et al.). The findings revealed the functional connectivity between dorsolateral prefrontal cortex and anterior cingulate cortex, and right dorsolateral prefrontal cortex and fronto-insular cortex using spectral dynamic causal modeling. The connectivity of these regions governed working memory ability and differences in resting-state effective connectivity might explain individual differences in working memory ability.

It has been revealed that speaking more than one language protects the executive functions in ageing. It is assumed that inhibition of one language whilst engaged in the other language confers an interference suppression advantage in bilingual individuals. However, available evidence also indicates that any executive control advantage is offset by poorer ability on language-specific tasks in bilinguals when compared to monolinguals. Kousaie et al. investigated the purported bilingual advantage on inhibitory control tasks in monolingual Anglophones and Francophones, and French/English bilingual young and older adults. Their findings did not show the expected bilingual advantage for executive task performance (except for a slight bilingual advantage on one measure), or a bilingual disadvantage on language tasks when compared to monolinguals. One possible explanation of findings is the mediating effect of context and frequency of exposure to both languages on executive and language skills in bilinguals, indicating that purported cognitive advantage/and disadvantage warrants further investigation, particularly in relation to resilience associated with ageing.

There is an extensive literature on the role of executive function deficits to socio-cognitive behavioral problems, yet there is no overarching theoretical framework that delineates how executive deficits impact social functioning. Wood and Worthington reviewed the literature on the purported link between executive function and socio-emotional functions in healthy ageing and post-traumatic brain injury (TBI) populations. They concluded that intact executive ability is crucial for appraisal and evaluation of social stimuli, and that the distinction between cognitive and socioemotional sequelae of TBI is no longer tenable based on current evidence. Torske et al. investigated whether social function problems were associated with specific executive impairments in children and adolescents with a diagnosis of Autism Spectrum Disorder (ASD) using parent-rated measures. Metacognitive executive functions contributed to social ability in young people with ASD and impaired social functioning potentially reflected poor behavioral regulation.

Jasinska argued that it is time to reconsider whether inhibitory executive processes are conceptually different from response selection and execution. The evidence from neuroscience accounts of inhibitory control mechanisms indicate that response inhibition could be simultaneously classified as a control process *and* a prepotent response tendency. This conceptualization provides a potential new approach to treating disorders of inhibitory control. Inhibitory control was also poor in those with chronic neuropathic or radicular pain when compared to controls that may have been caused by the chronic experience of pain or secondary tendency to have higher anxiety and depression levels than controls (Moriarty et al.). Problem solving executive ability was not affected by chronic pain in this cohort indicating selective effects of pain on cognition.

The measurement of executive functions remains problematic because (i), current standardized tests are not *process pure*: supervisory, attentional, and control executive functions invariably operate across other lower-level functions and (ii), there are abiding issues of sensitivity and ecological validity with current standardized tests. To address these issues, a computerized cooking task was developed to evaluate whether an analogue of real world behavior requiring multiple executive functions reliably indexed ability in a control group when compared to standard neuropsychological measures (Doherty et al.). Task parameters distinguished executive ability from overall IQ, unlike several currently widely used measures, and difficult task levels tapped different executive and memory functions when compared to easier levels. Test analogues of real-world tasks are potential candidates for next generation executive function measures. Tanguay et al. investigated executive function ability using a Breakfast Task, an Activities of Daily Living scale, and real cooking activity with acquired brain injury patients and matched controls. Patients had significant problems with all aspects of the Breakfast Task when compared to controls, although *real* cooking activity did not correlate with task performance indicating that purported ‘real world’ tasks may not isolate and capture the same functions used in everyday behavior. McGuire commented on the importance of developing ecologically valid tests of executive function for clinical assessment but also emphasized the multi-sensory context of real world behavior, which at present cannot be captured in immersive or computerized tasks but remains a possibility for the future. Cipresso et al. measured executive function ability in Parkinson's disease patients with and without cognitive impairments and controls on a virtual version of the Multiple Errands test (VMET) and standardized executive tests. Patients made more errors on the VMET than controls and the task was more sensitive to detection of early executive deficits than standardized executive measures. Sensitivity and reliability were also problematic on another widely used SELF and OTHER rating scale of executive ability. McGuire et al. compared Self, Other, and clinician ratings on the dysexecutive questionnaire to investigate factor structure and inter-rater reliability of patients' ratings of deficits when compared to others' ratings of their problems. There was poor agreement between clinician and other ratings on the measure indicating that accurate reporting of patients' post-injury deficits was essential to maintain the reliability and usefulness of the scale. Other raters should be selected with caution when asked to rate patient's problems on the dysexecutive questionnaire. Overall findings presented in this research topic were promising for new ecologically valid measures of executive function but were less encouraging for current measures of executive function and their clinical utility.

Finally, new approaches to enhance executive function ability are emerging associated with exercise and athleticism. The *neural efficiency* theory hypotheses that brain activity is attenuated in experts when compared to non-experts due to processing efficiency. When athletes and non-athletes were compared on a visuospatial executive task, athletes had faster reaction time responses but were not more accurate than non-athletes (Guo et al.). FMRI data showed that athletes had reduced activation to multiple frontal, temporal, and cerebellar regions during task performance when compared to non-athletes indicating task-specific neural reorganization in experts. Young healthy males were assigned to either high-, moderate-intensity or a no-exercise group and cortisol levels, EEG activity and executive function performance were measured (Liang Tsai et al.). Changes were seen in cortisol and P3 amplitude levels after resistance exercise along with enhanced executive function performance. The neural bases of these changes need further investigations, but effects may be associated with physiological arousal levels. Active participants also had better verbal working memory capacity, dual-task performance, and inhibitory ability when compared to a sedentary group (Padilla et al.). The authors concluded that chronic aerobic exercise can benefit cognitive and physical health across the lifespan.

In other work, Tsai et al. investigated the effects of resistance exercise on executive function performance in healthy elderly males and controls, and measured insulin, growth hormone and homocysteine levels at baseline and 1-year intervention period. Performance improvements in the exercise group were associated with an increase in growth factor levels, improved P3 amplitudes (indicating better attention), and decreased serum homocysteine. Aside from the physical benefits, regular consistent exercise contributed to improved executive function and general cognitive health in a well elderly cohort.

Collectively, the papers comprising this special topic reveal that executive function research is theoretically and methodologically diverse cross-cutting atypical, typical, and ageing populations. The neural bases of executive functions and working memory are receiving renewed interest using innovative and advanced imaging and modeling approaches. Importantly, new generation executive tests are emerging, that whilst in the early stages offer promise for speedy, sensitive process-specific clinical assessment. Finally, the relationship between cognitive and physical health reveals that healthy ageing need not be a process of gradual decline and executive functions, like cognition; generally, can be improved by enhanced physical health. Together these findings will hopefully stimulate new theoretical approaches and advances in the field.

## Author contributions

LB and NM wrote the editorial and were editors of the research topic.

### Conflict of interest statement

The authors declare that the research was conducted in the absence of any commercial or financial relationships that could be construed as a potential conflict of interest.
